# *K*_D_ determination from time-resolved experiments on live cells with LigandTracer and reconciliation with end-point flow cytometry measurements

**DOI:** 10.1007/s00249-021-01560-2

**Published:** 2021-07-24

**Authors:** Diana Spiegelberg, Jonas Stenberg, Pascale Richalet, Marc Vanhove

**Affiliations:** 1 Department of Surgical Sciences, Uppsala University, 751 85 Uppsala, Sweden; 2 Ridgeview Instruments AB, Skillsta 4, 740 20 Vänge, Sweden; 3 Present Address: A3P Biomedical AB, Vallongatan 1, 752 28 Uppsala, Sweden; 4 BioRevera, LLC, Scarborough, ME 04074 USA; 5 Marc Vanhove Consultancy, 4100 Boncelles, Belgium; 6 Present Address: Oxurion N.V., Gaston Geenslaan 1, 3001 Leuven, Belgium

**Keywords:** LigandTracer, Flow cytometry, Affinity, Binding kinetics, Live-cells measurements

## Abstract

Design of next-generation therapeutics comes with new challenges and emulates technology and methods to meet them. Characterizing the binding of either natural ligands or therapeutic proteins to cell-surface receptors, for which relevant recombinant versions may not exist, represents one of these challenges. Here we report the characterization of the interaction of five different antibody therapeutics (Trastuzumab, Rituximab, Panitumumab, Pertuzumab, and Cetuximab) with their cognate target receptors using LigandTracer. The method offers the advantage of being performed on live cells, alleviating the need for a recombinant source of the receptor. Furthermore, time-resolved measurements, in addition to allowing the determination of the affinity of the studied drug to its target, give access to the binding kinetics thereby providing a full characterization of the system. In this study, we also compared time-resolved LigandTracer data with end-point *K*_D_ determination from flow cytometry experiments and hypothesize that discrepancies between these two approaches, when they exist, generally come from flow cytometry titration curves being acquired prior to full equilibration of the system. Our data, however, show that knowledge of the kinetics of the interaction allows to reconcile the data obtained by flow cytometry and LigandTracer and demonstrate the complementarity of these two methods.

## Introduction

Cell surface receptors, including—but not limited to—G-protein-coupled receptors (GPCRs), constitute the most successful class of target proteins for drug discovery research (Cacace et al. 2003). Quantitative measurement of binding affinity is key to searching for and characterizing new receptor–ligand pairs for drug development. The equilibrium dissociation constant *K*_D_ describes the interaction of a drug or ligand *L* with a receptor *R* (with *K*_D_ = [*L*].[*R*]/[LR]) and is a critical metric to characterize the binding affinity of ligand–receptor interactions. *K*_D_ values are generally either obtained directly from equilibrium titration curves or deduced from either the ratio of the binding kinetics *k*_off_ (dissociation rate constant) and *k*_on_ (association rate constant) or from the free energy of binding ∆*G*_binding_ (Hulme and Trevethick 2010; Pan et al. 2013; Pollard 2010).

In recent years, in addition to *K*_D_ determination, special emphasis has been given to methodologies that also give access to the binding kinetics, thereby allowing a description of the entire dynamic aspect of ligand–receptor interactions for the prospective design of drugs with improved safety and efficacy profiles, and with better defined binding mechanisms (Andersson et al. 2006; Copeland et al. 2006; Dahl and Akerud 2013; Georgi et al. 2018; Schreiber 2002; Swinney 2008, 2009).

Paralleling the rise of biophysical methods in drug discovery, biophysical instrumentation has also in recent years improved in speed, sensitivity, robustness, and dynamic range, delivering rigorous, reliable, high-definition, and information-rich data. This field is constantly evolving to improve the drug discovery process and to address the new challenges of the next-generation therapeutics (Cariuk et al. 2013; Renaud et al. 2016; Santiveri et al. 2017).

For example, over the past two decades, the need for higher specificity drugs with improved in vivo efficacy and better alignment with physiology has led to a quest for tight binding pairs with sub-nanomolar to picomolar *K*_D_’s (Graff and Wittrup 2003; Rathanaswami et al. 2005; Schreiber 2002; Selzer et al. 2000). As a result, the low concentrations used for these types of assays require sensitive instrumentation. Furthermore, low concentrations and slow dissociation rates result in times to equilibrium that can span days, often exceeding the period of stability of the studied systems and making end-point *K*_D_ measurements difficult if not impossible (Andersson et al. 2010; Drake et al. 2018; Vanhove and Vanhove 2018). *K*_D_ measurements are also complicated in techniques, such as flow cytometry when *R*_0_ » *K*_D_ (with *R*_0_ as the total receptor concentration), leading to *R*_0_-driven interactions, where the value of *K*_D_ is easily misinterpreted (Drake and Klakamp 2007; Tamaskovic et al. 2012; Vaish et al. 2020). Pitfalls for proper *K*_D_ measurements of tight binders extend to time-resolved techniques even though they do not require equilibrium to be reached: dissociation rates slower than 10^–5^ s^−1^ generally involve several hour-long dissociations requiring stable baselines and rigorous assay settings, often pushing instrumentations to their limits (Barta et al. 2014; Jonsson et al. 2008; Rich and Myszka 2009). The growing trend for tight *K*_D_, therefore, needs to be better supported with methods and technologies that accurately measure these tight interactions (Drake et al. 2018).

Another biophysical challenge faced by the next-generation therapeutics relates to the fact that receptor targets may lose their 3D structure and/or function when isolated. For this reason, the measured *K*_D_ values of a therapeutic-target pair can vary widely depending on whether the receptor target is presented on live cells or expressed as a recombinant protein (Cariuk et al. 2013; Drake et al. 2018; Nilvebrant et al. 2012; Rathanaswami et al. 2008). The discrepancy of *K*_D_ values can also extend to the same ligand–receptor pair among different cell lines, highlighting cell-dependent receptor landscapes and receptor subunit pairing (Barta et al. 2014; Bjorkelund et al. 2011). As a result, technologies and methods applicable to live cells strengthen the physiological relevance of biophysical measurements and are, for that reason, gaining in popularity (Bondza et al. 2017; Drake et al. 2018; Rathanaswami et al. 2008; Renaud et al. 2016; Wood et al. 2004).

LigandTracer is a relatively new technology that allows real-time monitoring of ligand–receptor interaction on live cells (Bjorke and Andersson 2006; Bondza et al. 2017). Similar to other time-resolved techniques, where one molecular partner is immobilized while the other is added to the medium, the resulting time-traces represent the number of ligand–receptor (LR) complexes forming or dissociating over time and thus contain the kinetic information characterizing the studied molecular interaction (Canziani et al. 2004; Dubois et al. 2013). Binding kinetics are extracted from global fitting of the curves, and the value of *K*_D_ can be calculated from the ratio of the binding kinetics without the need to reach equilibrium. The use of live cells exposing native receptors circumvents the need for recombinant targets. Furthermore, LR complex dissociation can be monitored for several hours making it suitable for the characterization of slowly dissociating drugs. Here we report LigandTracer data for five tight therapeutic antibody–receptor pairs to illustrate the potential of the technology.

Furthermore, we compare LigandTracer kinetic data with traditional endpoint-based flow cytometry data. Flow cytometry-based *K*_D_ measurements also use live cells and involve the titration of a fixed number of cells with various concentrations of a fluorescent ligand for a specific time. The resulting curve, representing the amount of LR complex formed as a function of the ligand concentration, is typically analyzed based on a 1:1 equilibrium model to extract the *K*_D_ value (Drake and Klakamp 2007; Tamaskovic et al. 2012; Vaish et al. 2020). This end-point technique relies on the hypothesis that the incubation time used in the experiment is sufficiently long to reach equilibrium. Our kinetic data suggest that this prerequisite can be difficult to achieve in practice. Instead, we propose a general methodology combining LigandTracer and flow cytometry measurements, the two methods complementing each other to provide the best and most accurate description of the studied system.

## Materials and methods

### Cell culture

Cell lines (all from ATCC) were cultured in a humidified incubator at 37 °C with 5% CO_2_. SKBR3 cells expressing HER2 were grown in ATCC-formulated McCoy’s 5a Medium Modified (ATCC, Manassas, VA), supplemented with 10% FBS (Millipore-Sigma, Burlington, MA). HER2-expressing ovarian carcinoma SKOV3 cell lines were cultured in Ham’s F10 medium (Biochrom AG, Berlin, DE) supplemented with 10% FBS (Millipore-Sigma, Burlington, MA), 1% l-glutamine, and 1% penicillin–streptomycin (Biochrom AG, Berlin, DE). Daudi cells were cultured in RPMI 1640 cell culture medium (Biochrom AG, Berlin, DE) with the same supplements as above.

### Antibodies and labeling

Trastuzumab (Apoteket, Sweden), Pertuzumab (Omnitarg™; Genentech, South San Francisco, CA), Rituximab (Apoteket, Sweden), Cetuximab (Apoteket, Sweden) were fluorescently labeled for LigandTracer and/or flow cytometry experiments. Trastuzumab was labeled with Mix-n-Stain™ CF™ 488A and Mix-n-Stain™ CF™ 647 Antibody Labeling Kits (Millipore-Sigma, Burlington, MA) for LigandTracer and flow cytometry experiments, respectively, according to the manufacturer’s recommendations. Rituximab, Pertuzumab, and Cetuximab were labeled with fluorescein isothiocyanate (FITC) (Millipore-Sigma, Burlington, MA) for flow cytometry experiments. FITC was dissolved at 1 µg/µL in DMSO. Antibodies in PBS were diluted in twice the volume of borate buffer pH 9, and 100 ng of FITC was added per µg of antibody. The samples were incubated at 37 °C for 90 min. Labeled proteins were purified by buffer exchange on NAP-5 columns (GE Healthcare) and stored in aliquots at  − 20 °C prior to use (Bondza et al. 2017).

### LigandTracer

SKBR3 cells were lifted with Accutase^®^ (Millipore-Sigma, Burlington, MA), counted, and re-suspended at a density of 3.3 × 10^5^ cells/mL in full medium. Three milliliters of the cell suspension were seeded to tilted cell culture treated Petri dishes (Nunc #150350, ThermoFisher, Waltham, MA) and allowed to adhere to the plastic for 4 h at 37 °C after which the medium was replaced by 12 mL of fresh medium and the plates were incubated overnight horizontally. LigandTracer^®^ experiments were conducted 2–3 days after seeding.

Binding of Trastuzumab-A488 to SKBR3 cells was measured with LigandTracer Green (Ridgeview Instruments AB, Uppsala, Sweden) using a blue (488 nm)–green (535 nm) detector. Experiments were performed essentially as previously described (Bondza et al. 2017). In short, a Petri dish with adherent cells in a confined area and 3 mL of fresh culture medium with 0.1% sodium azide (Millipore-Sigma) to prevent internalization was placed on an inclined, rotating support in the instrument. After 30 min of baseline measurement, two increasing concentrations of a fluorescently labeled antibody were added sequentially and signals from cell target and background reference areas were recorded over time. Each sample was incubated for 2–5 h until sufficient curvature was obtained. Antibody dissociation from target was measured for 9 h after replacement of the incubation solution with fresh medium either in the absence or in the presence of an excess of unlabeled antibody. Kinetic traces were analyzed with TraceDrawer (Ridgeview Instruments AB, Uppsala, Sweden) using either a standard one-to-one binding model (referred to as “OneToOne model” in TraceDrawer, and essentially similar to Eq. 7 below) or, for situations where the number of ligand molecules is close to the number of receptors, a binding model corrected for ligand depletion (referred to as “OneToOneDepletionCorrected model” in TraceDrawer, and essentially similar to Eq. 2 below).

Experimental conditions to obtain LigandTracer kinetic data for Rituximab, Pertuzumab, Cetuximab, and Panitumumab were described elsewhere (Bondza et al. 2017; Barta et al. 2014).

### Flow cytometry

SKBR3 cells were re-suspended in 10 mL of full medium after harvest, counted, and allowed to recover for 1 h at 37 °C. Although it is unclear from the literature whether Trastuzumab remains at the cell surface or internalizes (Ram et al. 2014), cells were spun down and re-suspended at 1 × 10^6^ cells/mL at room temperature in full medium containing 0.1% sodium azide to prevent internalization. A total of 2.0 × 10^5^ cells in 200 µL were added per well of round bottom polypropylene 96-well plates (USA scientific). Plates were spun down at 4000 rpm for 4 min and flipped down vigorously once. Twenty-one concentrations of Trastuzumab-CF 647 ranging from 0.02 to 150 nM in full medium containing 0.1% sodium azide were added per well, in triplicate, for 3.5 h at room temperature and under gentle rocking. Plates were then spun down and fresh medium was added. The gate on live cells population was taken from a control cell well (without any antibody) and Guava^®^ easyCyte benchtop instrument (Millipore-Sigma) was set up to acquire 5000 events.

SKOV3 cells were re-suspended in 10 mL full of medium after harvest, counted, and allowed to recover for 1 h at 37 °C in full medium. Cells were then spun down (4000 rpm, 4 min) and re-suspended at a concentration of 1 × 10^6^ cells/mL. Cells were added to an antibody titration series of 0.07–100 nM and incubated in the dark for 2 h at room temperature under gentle rocking followed by analysis using a BD LSRII SORP (Becton Dickinson Biosciences, San Jose, USA) flow cytometer. The gate on live cells population was taken from a control cell well (without any antibody) and 10,000 events were collected.

## Equations used

In its simplest form, binding of a ligand *L* to its cognate receptor *R* can be written as Eq. 1, where *k*_on_ and *k*_off_ are the association and the dissociation rate constants, respectively:

1 $$ L\, + \;R \; \mathop \rightleftarrows \limits_{{k_{{{\text{off}}}} }}^{{k_{{{\text{on}}}} \,}} \,{\text{LR}}. $$

The differential equation describing [LR] versus time can be expressed as Eq. 2, where *L*_0_ and *R*_0_ are the total concentration of *L* and *R*, respectively, and [LR]_*t*_ is the concentration of the complex LR at time *t*:

2 $$ \frac{{\partial \left[ {{\text{LR}}} \right]_{t} }}{{\partial t}}\, = \,k_{{{\text{on}}}}  \cdot \left( {R_{0}  - \left[ {{\text{LR}}} \right]_{t} } \right) \cdot \left( {L_{0}  - \left[ {{\text{LR}}} \right]_{t} } \right) - k_{{{\text{off}}}}  \cdot \left[ {{\text{LR}}} \right]_{t} . $$

The integrated form of Eq. 2 was recently proposed by Vanhove and Vanhove (2018) in the form of Eq. 3, which allows to express [LR]_*t*_ as a function of *k*_on_, *k*_off_, *R*_0_, *L*_0_, and *t*:

3 $$ \left[ {{\text{LR}}} \right]_{t}  = \frac{{a \cdot \left( {1 - c} \right) - b \cdot \left( {1 + c} \right)}}{{2 \cdot k_{{{\text{on}}}}  \cdot \left( {1 - c} \right)}} $$

where $$a = k_{{{\text{on}}}}  \cdot \left( {L_{{\text{0}}}  + R_{{\text{0}}} } \right) + k_{{{\text{off}}}};\;b = \sqrt {a^{2}  - 4 \cdot k_{{{\text{on}}}}^{2}  \cdot L_{{\text{0}}}  \cdot R_{{\text{0}}} };\;c = \left( {\frac{{a - b}}{{a + b}}} \right) \cdot e^{{ - b \cdot t}}.$$

The experimental signal (*S*)_*t*_, whether in flow cytometry or LigandTracer experiments, is directly proportional to the concentration of complex LR and can thus be expressed as Eq. 4, where *S*_max_ is the maximum signal, i.e., the signal at saturating ligand concentrations, and where [LR]_*t*_ is given by Eq. 3:

4 $$ \left( S \right)_{t} \, = \,S_{{{\text{max}}}}  \cdot \frac{{\left[ {LR} \right]_{t} }}{{R_{0} }}. $$

Equation 4 allows the analysis of pre-equilibrium titration curves. Of note, Eq. 3 is derived without any mathematical simplification and is thus not limited to pseudo first-order reactions, where *L*_0_ >  > *R*_0_ (Vanhove and Vanhove 2018).

At *t* = ∞, *c* = 0 and Eqs. 3 and 4 can be simplified into Eqs. 5 and 6, respectively, where [LR]_*e*_ is the concentration of complex LR at equilibrium (Vanhove and Vanhove 2018):

5 $$ \left[ {LR} \right]_{e} \, = \,\left( {\frac{{L_{{\text{0}}}  + R_{{\text{0}}}  + K_{{\text{D}}} }}{2}} \right)\,\, - \,\sqrt {\left( {\frac{{L_{{\text{0}}}  + R_{{\text{0}}}  + K_{{\text{D}}} }}{2}} \right)^{2}  - L_{{\text{0}}}  \cdot R_{{\text{0}}} } $$

6 $$ \left( S \right)_{t} \, = \,S_{{\max }}  \times \frac{{\left[ {LR} \right]_{e} }}{{R_{0} }}. $$

Finally, when *L*_0_ >  > *R*_0_, Eq. 2 can be simplified into Eq. 7 which, following a reasoning similar as above, leads to Eq. 8 (Morton et al. 1995):

7 $$ \frac{{\partial \left[ {LR} \right]_{t} }}{{\partial t}}\, = \,k_{{{\text{on}}}}  \cdot \left( {R_{0}  - \left[ {LR} \right]_{t} } \right) \cdot L_{0} \, - \,k_{{{\text{off}}}}  \cdot \left[ {LR} \right]_{t} $$

8 $$ \left( S \right)_{t} \, = \,S_{{{\text{max}}}} .\left( {\frac{{L_{0} }}{{L_{0}  + K_{{\text{D}}} }}} \right).\left( {1 - e^{{ - \left( {k_{{{\text{on}}}} .L_{0}  + k_{{{\text{off}}}} } \right) \cdot t}} } \right). $$

## Data analysis

**Fig. 1:**
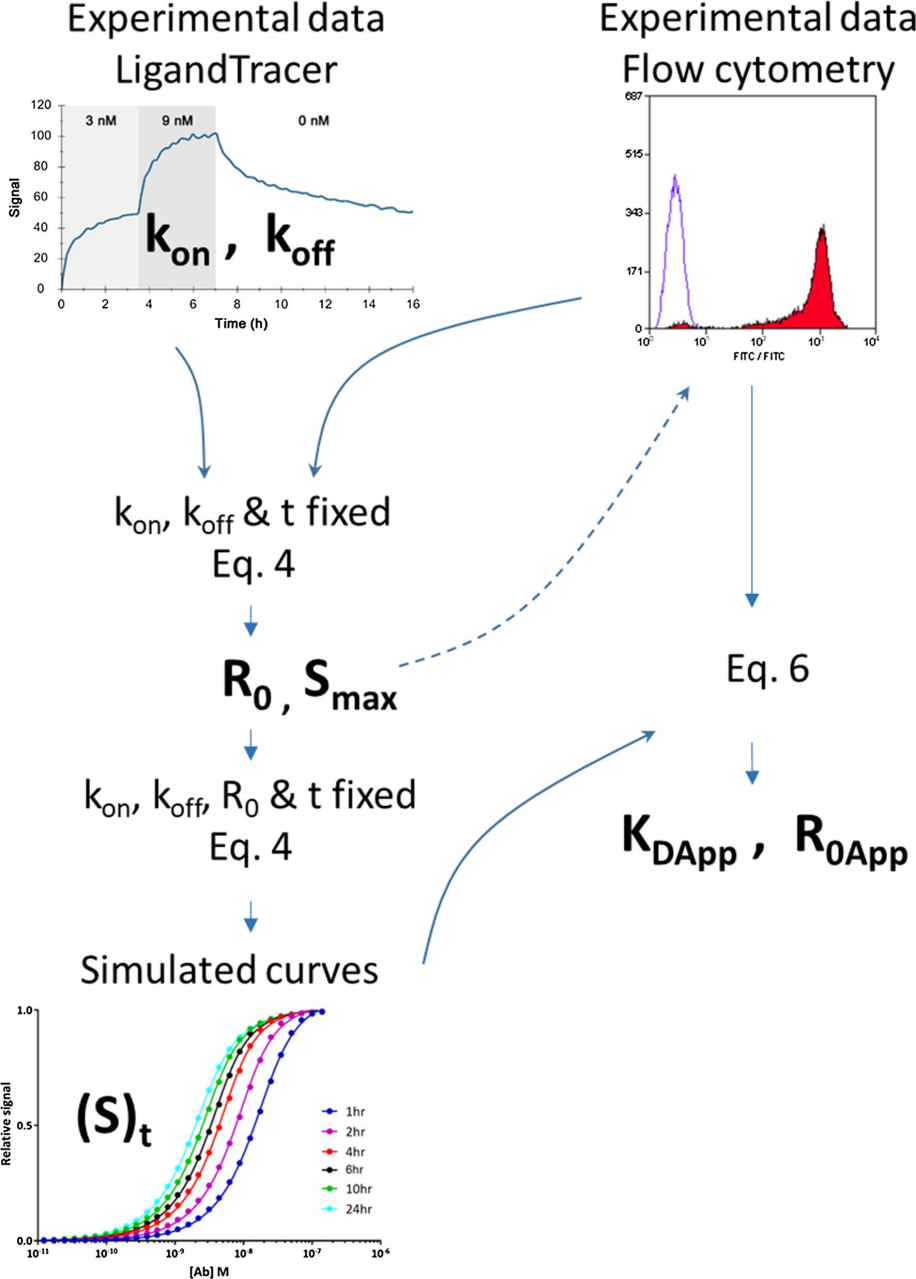
Overview of data analysis workflow. Bold characters indicate calculated variable values. Experimental LigandTracer data were used to calculate the kinetic constants *k*_on_ and *k*_off_. Experimental flow cytometry data were then fitted to Eq. 4 with binding kinetics obtained from LigandTracer and incubation time as constants, giving access to the receptor concentration term *R*_0_. Binding kinetics and *R*_0_ values were used in Eq. 4 to simulate flow cytometry data at various incubation times, allowing a visual assessment of the progress of the reaction towards equilibrium. Both simulated and experimental flow cytometry data were then analyzed with Eq. 6 to calculate the apparent equilibrium dissociation constant (*K*_DApp_) and apparent receptor concentration (*R*_0App_)

An overview of the data analysis workflow is shown in Fig. 1.

LigandTracer data were analyzed with TraceDrawer to compute *k*_on_ and *k*_off_ values. When shown, the *U *value was used to measure the precision of the fits and defines how much a given kinetic parameter can vary before the results show significant changes. The lower the *U *value, the better the fit. A *U *value > 15 for example indicates that the binding kinetics can be altered by 15% or more without significantly affecting the fitted curve. The *U *value takes parameter dependency into account and is, therefore, a safer quality value than χ^2^ or *T *values (Onell and Andersson 2005).

Experimental flow cytometry data were first fitted with Eq. 4 with the help of the GraphPad Prism software (GraphPad Software Inc., La Jolla, CA) using the *k*_on_ and *k*_off_ values obtained from LigandTracer experiments and fixing the term t to the actual incubation time, which allowed the determination the parameters *R*_0_ and *S*_max_. The number of receptors per cell was then calculated from the Avogadro number and the number of cells used in the experiment. For A431 cells, since experimental flow cytometry data for Panitumumab–EGFR binding were not generated, the value of *R*_0_ was taken from Barta et al. (2011) and fixed to 2 × 10^6^ receptors/cell. Equation 4 was then used to simulate flow cytometry curves at different incubation time based on the value of *S*_max_, *R*_0_, *k*_on_, and *k*_off_ obtained as described above.

In a second stage, flow cytometry data (normalized based on *S*_max_ to facilitate visual comparison) were fitted to Eq. 6 (using the Graphpad Prism software) without constraints. Since it was a priori not known whether the system was fully at equilibrium when the data were acquired, fitting with Eq. 6 yielded only apparent values for *K*_D_ and *R*_0_ which will be referred to here as and *K*_DApp_ and *R*_0App_.

Finally, time to equilibrium for a given ligand concentration was calculated from the ratio of Eqs. 3 and 5, the [LR]_*t*_/[LR]_*e*_ ratio representing the degree of completion of the reaction at a given time. A ratio of 1 indicates that the equilibrium has been reached.

## Results

### Binding kinetics with LigandTracer

**Fig. 2:**
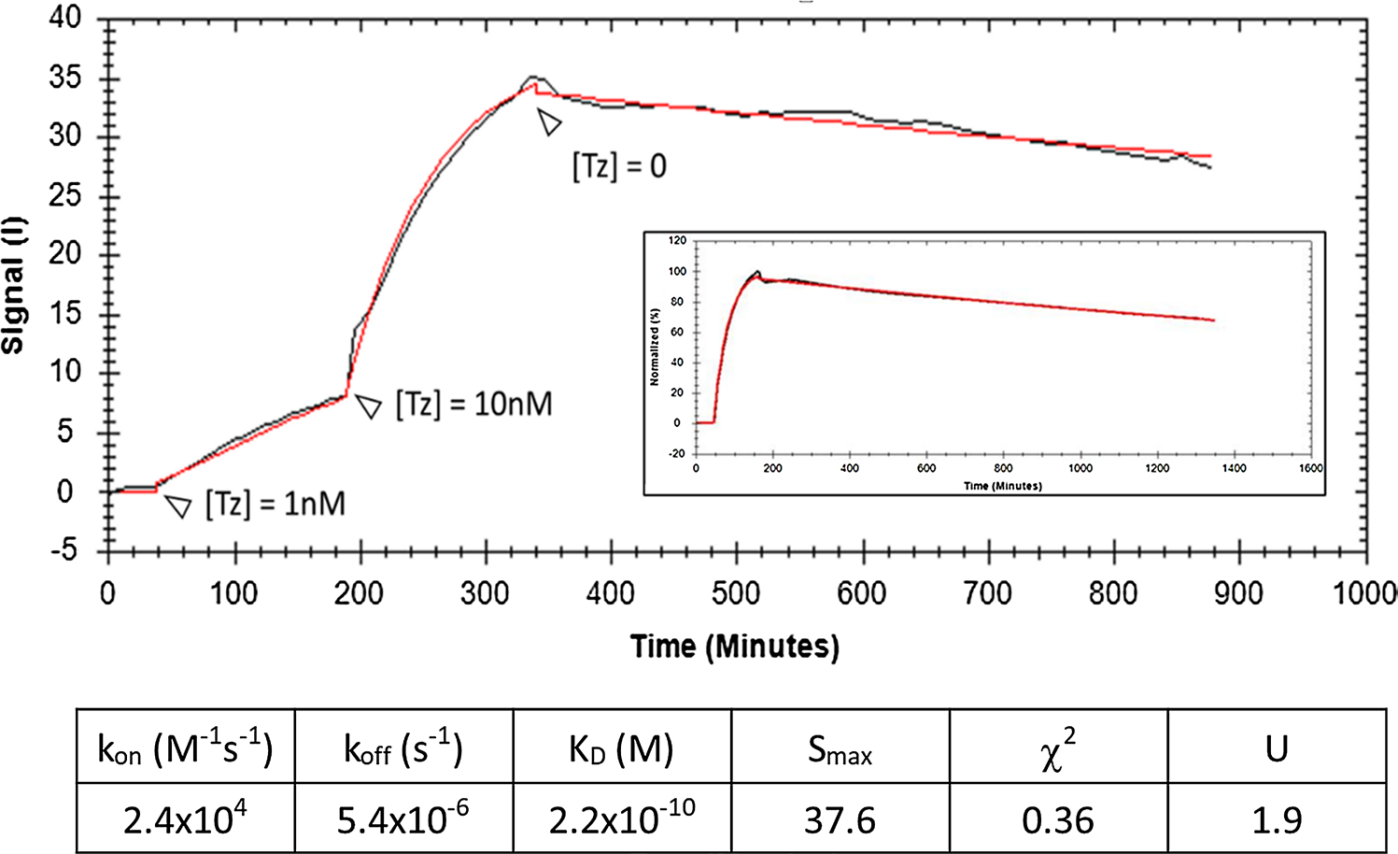
Time course (signal intensity vs*.* time) for binding of Trastuzumab–A488 (Tz) to SKBR3 cells. Two antibody concentrations of 1 and 10 nM were added sequentially for 150 and 200 min, respectively, and dissociation was followed for 9 h. The fitted curve for a 1:1 binding model is shown in red. Calculated values and fitting parameters are reported in the table below the curve. The insert shows the dissociation of Trastuzumab–A488 in the presence of 10 nM unlabeled antibody ([Tz] = 10 nM for the association phase)

Except for Trastuzumab binding to HER2 on SKBR3 cells, binding kinetics have been reported elsewhere (see data with respective references in Table 1). LigandTracer data for Trastuzumab are shown in Fig. 2. The kinetic traces could be accurately analyzed with a standard 1:1 binding model with the help of the TraceDrawer software with low χ^2^ and *U *values of 0.36 and 1.90, respectively, leading to *k*_on_, *k*_off_, and calculated *K*_D_ values of 2.4 × 10^4^ M^−1^ s^−1^, 5.4 × 10^–6^ s^−1^, and 2.2 × 10^–10^ M, respectively. Data analysis using a binding model corrected for ligand depletion yielded identical parameters. To be noted, and despite a dissociation rate < 10^–5^ s^−1^, the dissociation phase showed a decrease of 14%, allowing robust *k*_off_ estimation (the experiment was repeated independently twice resulting in identical *K*_D_ values and binding kinetics parameters within 2% standard deviation—not shown). In addition, worth noting, following the dissociation of Trastuzumab-A488 from SKBR3 cells in the presence of an excess of unlabeled antibody (insert in Fig. 2) provided a *k*_off_ value of 4.7 × 10^–6^ s^−1^ in excellent agreement with the value reported above, which demonstrates that rebinding of the antibody during the dissociation phase can be neglected.

**Table 1:** Binding kinetics and *K*_D_ values from LigandTracer measurements for the indicated antibody-receptor pairs, and receptor concentrations (*R*_0_) computed from flow cytometry data

Systems studied		Binding kinetics from LigandTracer				Flow cytometry, Eq. 4 with kinetic constants from LigandTracer		Flow cytometry, Eq. 6		Receptor density (million per cell)		
Drug-target	Cell type	*k*_on_ (M^−1^ s^−1^)	*k*_off_ (s^−1^)	*K*_D_ (M)	Reference	*R*_0_ (M)	*R*_0_/*K*_D_	*K*_DApp_ (M)	*R*_0App_ (M)	This paper	Literature	Reference
Rituximab-CD20	Daudi	1.4 × 10^4^	1.3 × 10^–5^	9.3 × 10^–10^	Bondza et al. (2017)	5.0 × 10^–9^	5.3	3.9 × 10^–9^	2.2 × 10^–8^	3	0.13–0.21	Bondza et al. (2020)
Trastuzumab-HER2	SKBR3	2.4 × 10^4^	5.4 × 10^–6^	2.2 × 10^–10^	This paper	9.9 × 10^–9^	45	1.7 × 10^–9^	6.7 × 10^–9^	5.9	5–6	Barta et al. (2011)
Pertuzumab-HER2	SKOV3	5.5 × 10^4^	7.0 × 10^–6^	1.3 × 10^–10^	Bondza et al. (2017)	1.0 × 10^–8^	79	4.1 × 10^–10^	9.5 × 10^–9^	10	5–6	Barta et al. (2011)
Panitumumab-EGFR	A431	6.3 × 10^4^	1.6 × 10^–6^	2.5 × 10^–11^	Barta et al. (2014)	3.3 × 10^–9a^	130	NA	NA	NA	2–5	Barta et al. (2011)
Cetuximab-EGFR	SKOV3	9.0 × 10^5^	1.2 × 10^–6^	1.3 × 10^–12^	Bondza et al. (2017)	2.2 × 10^–9^	1700	8.4 × 10^–11^	2.3 × 10^–9^	1	0.1– 0.3	Barta et al. (2011)

The incubation times for flow cytometry measurements were 1 h for Rituximab, 2 h for Pertuzumab and Cetuximab, and 3.5 h for Trastuzumab. Receptor concentrations were also converted to receptor density on cells and compared to published data. Finally, *K*_DApp_ and *R*_0App_ values obtained by analysis of flow cytometry data assuming equilibrium are also presented

*NA* data not available

^a^Except for Panitumumab-EGFR, for which the *R*_0_ value was obtained from Barta et al. (2011)

### Flow cytometry

**Fig. 3:**
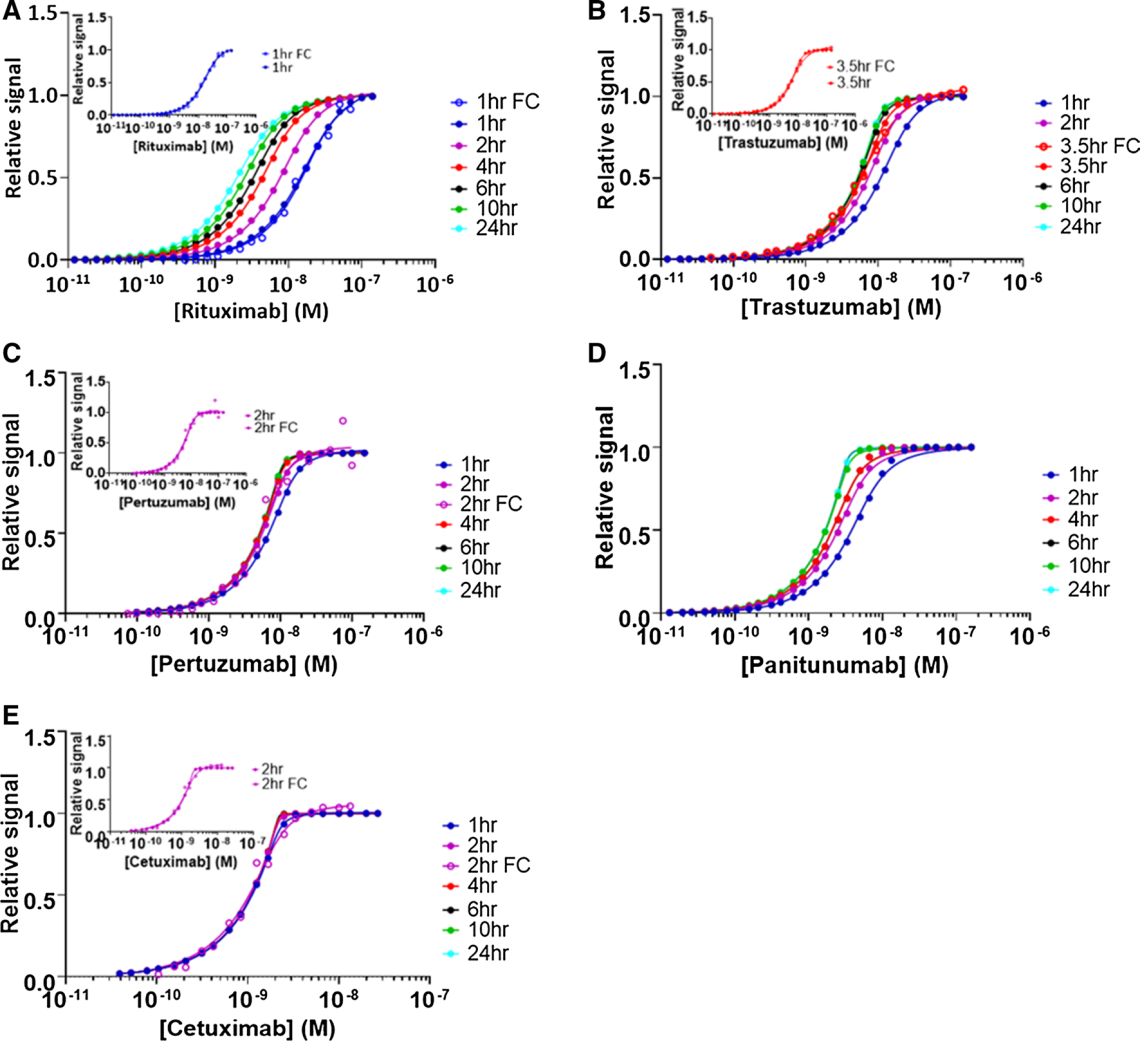
Comparison of normalized experimental flow cytometry (FC) data recorded at the indicated incubation time (average from duplicate or triplicate measurements) with flow cytometry data simulated for various incubation times with Eq. 4. **A** Rituximab–CD20 on Daudi cells, experimental flow cytometry incubation time 1 h with 10^6^ cells/mL; **B** Trastuzumab–HER2 on SKOV3 cells, experimental flow cytometry incubation time 3.5 h with 10^6^ cells/mL; **C** Pertuzumab–HER2 on SKOV3 cells, experimental flow cytometry incubation time 2 h with 6 × 10^5^ cells/mL; **D** Panitumumab–EGFR on A431 cells, only simulated flow cytometry data assuming 10^6^ cells/mL; **E** Cetuximab–EGFR on SKOV3 cells, experimental flow cytometry incubation time 2 h with 1.33 × 10^6^ cells/mL. The thumbnails in **A**, **B**, **C**, and **E** represent the experimental flow cytometry data together with the data simulated at the same incubation time

Experimental flow cytometry data for binding of Rituximab to CD20-expressing Daudi cells (incubation time 1 h), Trastuzumab and Pertuzumab to HER2-expressing SKOV3 cells (incubation time 3.5 and 2 h, respectively), and Cetuximab to EGFR-expressing SKOV3 cells (incubation time 2 h) are shown in Fig. 3. Worth mentioning, these incubation times, although selected arbitrarily, correspond to incubation times generally used for this kind of experiment, since longer incubation times are often associated with cell viability issues. For example, Freeman et al. (2012) performed flow cytometry titration experiments with Panitumumab on EGFR-expressing A431 cells with 1 h incubation time. We did not, however, assume that the different systems were necessarily at equilibrium at the indicated incubation times. Data analysis using Eq. 6 was thus not appropriate. Instead, we chose to fit the flow cytometry traces to Eq. 4, fixing the kinetic parameters *k*_on_ and *k*_off_ to the values obtained from LigandTracer experiments which allowed to extract the value of the receptor concentration (*R*_0_) for each system and cell type, from which the number of receptor molecules per cell was calculated. These values are reported in Table 1 and are consistent with published data for at least HER2-expressing SKBR3 and SKOV3 cells. For EGFR-expressing A431 cells, in the absence of flow cytometry data with Panitumumab, R_0_ was fixed to a value corresponding 2 × 10^6^ receptors/cell based on Barta et al (2011).

Equation 4 was then used to generate theoretical flow cytometry traces for the different systems and for various times of incubation (Fig. 3). From this, it is immediately evident for all studied systems except Cetuximab–EGFR that full equilibration requires incubation times significantly longer than 1 h, the Rituximab–CD20, Trastuzumab–HER2, and Panitumumab–EGFR systems being the slowest to equilibrate.

**Fig. 4:**
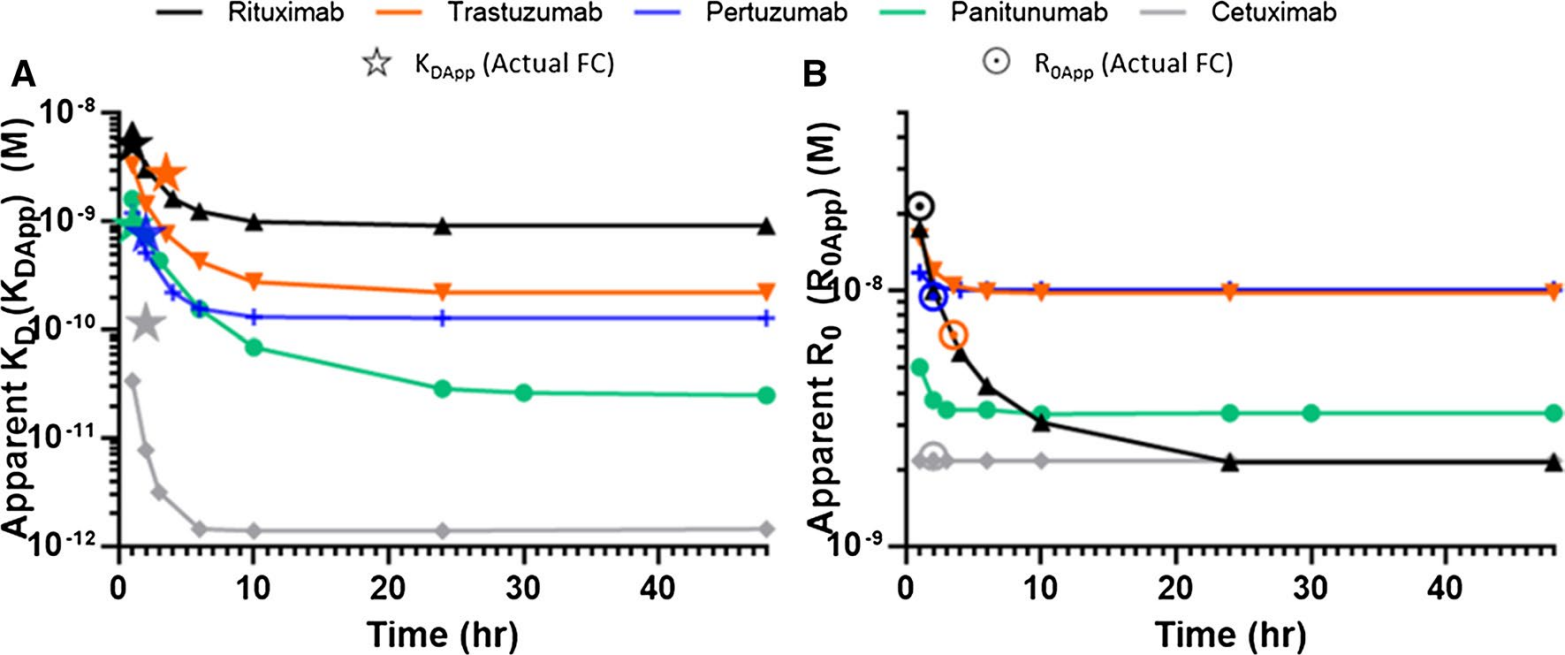
Experimental flow cytometry (FC) data as well as flow cytometry data simulated for various incubation times based on Eq. 4 were fitted with Eq. 6 to compute apparent equilibrium dissociation constants (*K*_DApp_) and apparent receptor concentrations (*R*_0App_). **A** *K*_DApp_ values. The stars correspond to *K*_DApp_ values obtained from the experimental flow cytometry data sets. **B** *R*_0App_ values. The dotted disks correspond *R*_0App_ values obtained from the experimental flow cytometry data sets

Flow cytometry experiments performed with the objective of determining the affinity of antibodies to cell surface receptors are generally analyzed using Eq. 6 assuming that the system is at equilibrium. The extent to which the computed parameters *R*_0_ and *K*_D_ deviate from their exact value when that latter hypothesis is not fulfilled can be appreciated by fitting either actual flow cytometry data sets acquired prior to equilibrium or theoretical simulated flow cytometry curves generated for different incubation times with Eq. 6 (Fig. 4A, B). This analysis reveals that *R*_0_ and *K*_D_ values computed prior to full equilibration (*R*_0App_ and *K*_DApp_) differ to a great extent from the intrinsic *R*_*0*_ and *K*_D_ values, even when visual observation of the curves suggests that the system is almost at equilibrium (see data for Cetuximab).

Altogether, these observations highlight the benefit of combining LigandTracer-based kinetic determination with flow cytometry experiments, especially for slowly equilibrating systems which may require incubation times far exceeding the time during which the reagents are stable to reach the equilibrium.

### Time to equilibrium

The time for a system to reach 97% of the final equilibrium has been described by Eq. 9 (Andersson et al. 2010; Hulme and Trevethick 2010):

9 $$ t_{{{\text{eq}}}} \, = \,\frac{{5~.~~ln2}}{{\left( {k_{{{\text{on}}}} ~.~~L~ + ~k_{{{\text{off}}}} } \right)}}~. $$

The above expression, however, assumes that the considered reaction obeys pseudo-first-order kinetics, which is true only if *L*_0_ >  > *R*_0_. To assess the degree of completeness of the reaction, we, therefore, propose to use the ratio of [LR]_*t*_ versus [LR]_*e*_ as obtained from Eqs. 3 and 5. The closer to 0, the further away the system is from equilibrium. Conversely, a ratio of 1 indicates that the system has reached equilibrium.

**Fig. 5:**
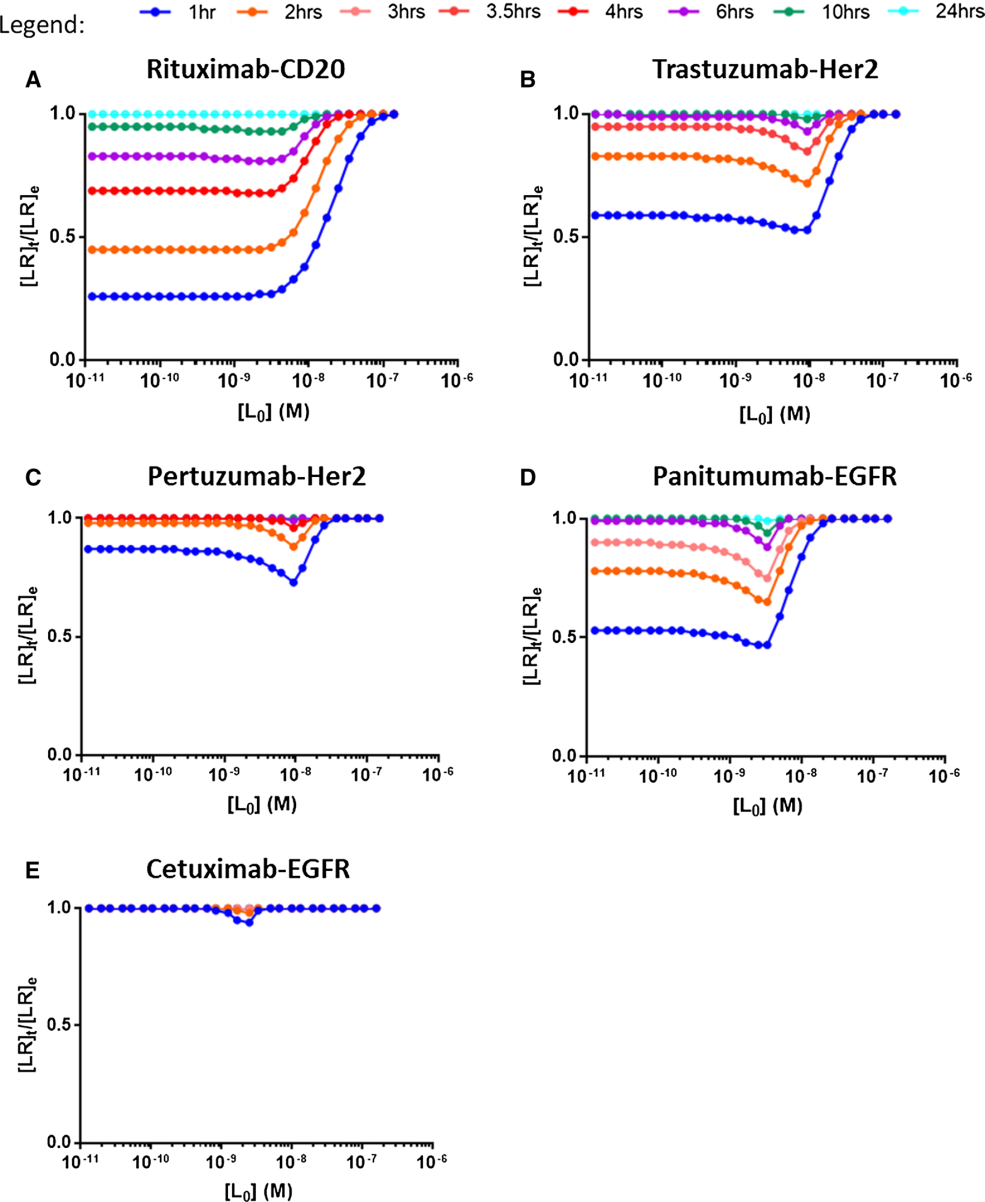
Equilibrium charts for the studied systems based on binding kinetics obtained from LigandTracer data and receptor concentrations obtained from flow cytometry data (all systems except Panitumumab) or literature (Panitumumab). The ratio of the concentration of the antibody–receptor complex at a given time ([LR]_*t*_, from Eq. 3) and at time infinite ([LR]_*e*_, from Eq. 5) was plotted against antibody concentration. At equilibrium, [LR]_*t*_ = [LR]_*e*_ and the [LR]_*t*_/[LR]_*e*_ ratio is equal to 1

Figure 5 represents plots of [LR]_*t*_/[LR]_*e*_ versus *L*_0_ for various incubation times for all the systems studied here, offering a visual assessment of time to equilibrium for all molecular pairs under their specific experimental settings. For example, the fast-equilibrating Cetuximab–EGFR and Pertuzumab–HER2 systems requires 1–3 h and 6–10 h for full equilibration, respectively, while the other systems have reached equilibrium for all ligand concentrations only after  ~ 24 h. It can also be appreciated that all considered systems are predicted to be at equilibrium for the highest ligand concentrations even for the shortest incubation time considered here, i.e., 1 h. Conversely, the time to equilibrium is longer for lower ligand concentrations. Interestingly, though, the value of [LR]_*t*_/[LR]_*e*_ reaches for all systems a minimum for *L*_0_ values roughly around 10^–9^–10^–8^ M as shown as a dip in the curves. Although counter-intuitive, this indicates that the ligand concentration that requires the longest time to reach equilibrium is not necessarily the lowest concentration used in an experimental setting. A formal mathematical demonstration of this observation would require calculating the *L*_0_ value for which the derivative of the [LR]_*t*_/[LR]_*e*_ expression (with [LR]_*t*_ and [LR]_*e*_ as per Eqs. 3, 5) is equal to zero, which is complex and far beyond the scope of this work. Instead, the numerical methodology proposed here allows to easily assess the time needed to reach the equilibrium for any pre-defined experimental conditions.

## Discussion

### LigandTracer information on kinetic constants to strengthen flow cytometry data

From Eq. 1, which describes the interaction between a ligand *L* and its receptor *R*, the rate of LR complex formation over time can be expressed without simplification as Eq. 2 or, assuming that *L*_0_ >  > *R*_0_, as Eq. 7. These are differential equations, which is the form usually used by software to globally analyze time-resolved binding data through numerical integration. Equation 2 (or variations thereof) is useful to analyze data generated under conditions where *L*_0_ ~ *R*_0_, and TraceDrawer is, to our knowledge, the only commercially available software offering it as an option.

Equation 4, or its simplified version (Eq. 8), are better suited to be used in software’s such as Excel for data simulation or GraphPad Prism for non-linear regression analysis. Eq. 4, derived from Eq. 3, and which can be used to describe or analyze pre-equilibrium titration curves, is, however, complex and may not be ideally suited to analyze only one set of flow cytometry data obtained for a specific incubation time. We, therefore, propose to use the kinetic parameters *k*_on_ and *k*_off_ obtained from LigandTracer experiments as constants to “stabilize” the equation and allow robust determination of the receptor concentration *R*_0_, as exemplified in this manuscript.

Fitting of flow cytometry data with Eq. 6 is only recommended if the system has reached full equilibrium. When that condition is not perfectly fulfilled, data analysis with Eq. 6 can return *R*_0_ and *K*_D_ values that can deviate substantially from the true values of these parameters depending not only on the degree of completion of the reaction but also, to a certain extent, on the relationship between *R*_0_ and *K*_D_ (Vaish et al. 2020).

The systems studied here can be sorted into three groups based on their respective *R*_0_/*K*_D_ ratio (Table 1), namely, (1) Rituximab–CD20 (*R*_0_ ~ *K*_D_); (2) Trastuzumab–HER2, Pertuzumab–HER2 and Panitumumab–EGFR (*R*_0_ > *K*_D_); and (3) Cetuximab–EGFR (*R*_0_ >  > *K*_D_). As discussed elsewhere (Drake et al. 2018; Drake and Klakamp 2007; Tamaskovic et al. 2012; Vanhove and Vanhove 2018), experimental data obtained under conditions where *R*_0_ ~ *K*_D_ allows the determination of both parameters, while only *R*_0_ can be robustly determined when *R*_0_ >  > *K*_D_. This also applies to analysis of pre-equilibrium titration curves with Eq. 4 (Vanhove and Vanhove 2018). Therefore, determination of *K*_D_ values from flow cytometry data without the support of information on the kinetics of the interaction such as provided by LigandTracer would have been challenging or impossible for all studied systems except Rituximab–CD20, and this regardless of whether the data would have been generated under conditions of pre-equilibrium and analyzed with Eq. 4 or under conditions of full equilibrium and analyzed with Eq. 6.

When conducting flow cytometry experiments with the objective of analyzing a given system at equilibrium, it is common practice to run the experiment at two (or more) incubation times and to consider in practice that the system has reached its equilibrium if the different data sets, e.g., in the form of saturation binding curves, superimpose. It is important to appreciate, however, that when *R*_0_ > *K*_D_ only a small fraction of the curve (corresponding to a narrow range of low ligand concentrations near the saturation plateau phase—see below) contributes to the numerical determination of *K*_D_. For that narrow range of concentration, equilibration will take longer than for most of the rest of the data sets and it is, therefore, easy to wrongly consider that the system is at equilibrium, while it is not, and, furthermore, for the most important part of the curve (see Fig. 3). This can be illustrated here from the Trastuzumab–HER2, Pertuzumab–HER2, and Cetuximab–EGFR flow cytometry data which, from a rough visual examination, seemingly appeared close to the equilibrium after incubation times of 3.5, 2 and 2 h, respectively. Yet, *K*_DApp_ values obtained from fitting the curves with Eq. 6 differed significantly from the real *K*_D_ values (Fig. 4).

**Fig. 6:**
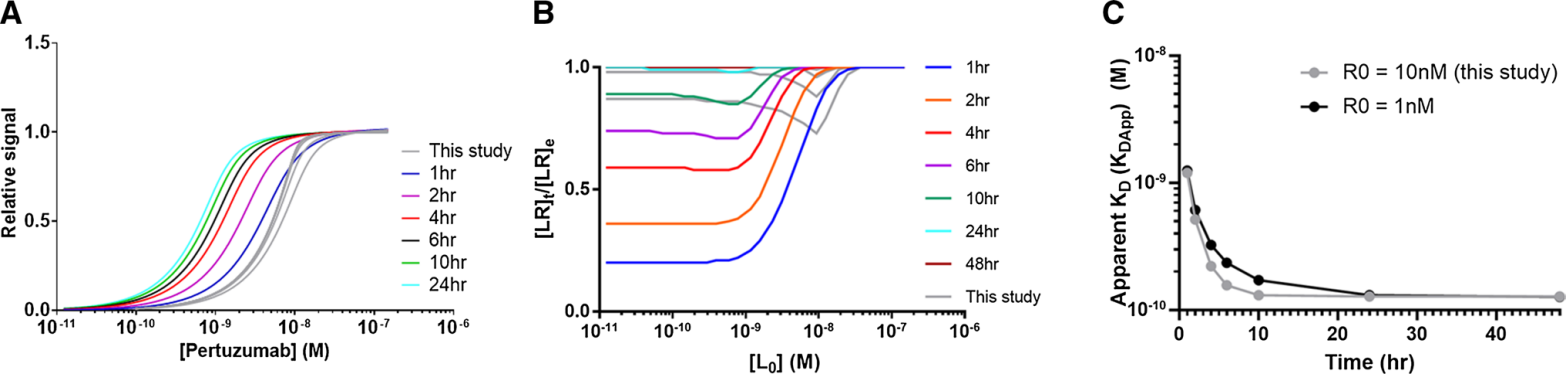
Influence of receptor concentration R_0_ on time to equilibrium and subsequent impact on *K*_DApp_ values exemplified by the Pertuzumab–HER2 system. A value of *R*_0_ = 1 nM was compared with actual *R*_0_ value (10 nM) used for experimental work in this study (light gray curves). **A** Simulated flow cytometry data (signal vs. antibody concentration for various incubation times). **B** Degree of advancement of the reaction towards equilibrium ([LR]_*t*_/[LR]_*e*_ versus antibody concentration [*L*_0_]). **C** Apparent *K*_D_ (*K*_DAPP_) vs. time of incubation

To explore this aspect further, we modeled, assuming unchanged kinetics, the Pertuzumab–HER2 interaction in an altered yet realistic setting, with *R*_0_ = 1 nM (vs. 10 nM), which could for example be achieved using a low concentration of BT474 cells which express less HER2 receptors than SKOV3. The data are reported in Fig. 6, with predicted flow cytometry traces, time to equilibrium data, and subsequent effects on *K*_DApp_ values compared to the setting used in this paper. Despite a marked difference in the predicted time to equilibrium (reached only after 24–48 h compared to 4–6 h for the current setting with SKOV3 cells), the difference in *K*_DApp_ for each given incubation time is minimum between the two conditions, as shown in Fig. 6A–C, illustrating the fact that attempts to reach the equilibrium faster by increasing R_0_ does not improve the robustness of affinity measurements on live cells.

It can also be appreciated from Fig. 6A that the shape of the curve changes significantly when increasing *R*_0_ from 1 to 10 nM, resulting in the fact that for the higher *R*_0_ value only a small part of the experimental data set, corresponding to the very top of the sigmoid curve, i.e., when a significant fraction of the receptor has been titrated, can be exploited to extract the value of *K*_D_. In practice, any experimental variability in that part of the curve will thus have strong repercussions on the computed *K*_D_ value. The Cetuximab–EGFR interaction studied here is an extreme example of an *R*_0_-driven system, i.e., of a system not allowing *K*_D_ determination from equilibrium experiments, because it is studied under conditions where *R*_0_ is much larger than *K*_D_.

Our recommendation to combine LigandTracer kinetic data with flow cytometry data, making no assumption on whether flow cytometry data are obtained at equilibrium or not, i.e., analyzing data with Eq. 4, eliminates all the risks described above and ensures robust determination of all the parameters describing the studied system, namely, *k*_on_, *k*_off_, *K*_D_, and *R*_0_, even under conditions where *R*_0_ is much larger than *K*_D_.

Finally, for researchers wishing to analyze flow cytometry data only under equilibrium conditions, the knowledge of the kinetic constants obtained from LigandTracer experiments will allow said researchers to determine, as shown here from [LR]_*t*_/[LR]_*e*_ versus *L*_0_ plots, the degree of completion of the reaction for any ligand concentration, and therefore, gain confidence that the experiment is conducted under proper conditions.

## Conclusion

Screening for biologics has evolved significantly in the past decades and often involves live cell receptors as some categories of targets can be difficult to express as recombinant proteins. Furthermore, new generation biologics frequently present very high affinities towards their cognate receptors which represents new challenges. LigandTracer measures the equilibrium dissociation constant *K*_D_ from the ratio of binding kinetics, thereby bypassing the need to reach equilibrium. It can also record dissociation for hours and measure off rates slower than 10^–5^ s^−1^, which are getting more frequent within this category of therapeutics. Finally, the technology uses live cells expressing functional receptors closer to in vivo conditions.

Flow cytometry measurements also involve live cells and titration with a labelled ligand to record molecular complex formation. However, measured *K*_D_ values do not always agree among those platforms. We hypothesized that these discrepancies, when they exist, originate most frequently from either flow cytometry experiments being recorded prior to full equilibration of the system or under conditions, where the receptor concentration is much larger than the value of the dissociation constant. Being operated under similar conditions, flow cytometry and LigandTracer platforms are thus ideal for cross-validation.

Ultimately, the goal of the drug discovery pipeline is to develop and produce safe and effective drugs. It is, therefore, essential to translate in vitro therapeutics–receptor interaction data in general, and *K*_D_ in particular, into hypothesis of biological effects. In vitro platforms are evolving, aiming to predict in vivo outcome such as described by Spiegelberg et al. (2016). Furthermore, joint analysis of binding kinetics, pharmacokinetics, target information, and dosage regimen during pharmacokinetic/pharmacodynamic modeling could be beneficial to early drug development (Georgi et al. 2018; Zhao and Schuck 2015). LigandTracer belongs to the arsenal of new tools now at the disposal of scientists to achieve these objectives.
